# Effects of animal protein supplementation of mothers, preterm infants, and term infants on growth outcomes in childhood: a systematic review and meta-analysis of randomized trials

**DOI:** 10.1093/ajcn/nqy348

**Published:** 2019-06-08

**Authors:** Laura Pimpin, Sarah Kranz, Enju Liu, Masha Shulkin, Dimitra Karageorgou, Victoria Miller, Wafaie Fawzi, Christopher Duggan, Patrick Webb, Dariush Mozaffarian

**Affiliations:** 1 Friedman School of Nutrition & Science Policy, Tufts University, Boston, MA; 2 Harvard T.H. Chan School of Public Health, Boston, MA; 3 Boston Children's Hospital, Boston, MA

**Keywords:** dietary protein, child, maternal, weight, height, anthropometric, birth weight, meta-analysis

## Abstract

**Background:**

Child stunting is a major public health problem, afflicting 155 million people worldwide. Lack of animal-source protein has been identified as a risk, but effects of animal protein supplementation are not well established.

**Objective:**

The aim of this study was to investigate effects of animal protein supplementation in mothers, preterm infants, and term infants/children on birth and growth outcomes.

**Methods:**

PubMed, EMBASE, Cochrane library, Web of Science, Cumulative Index of Nursing and Allied Health Literature, and Latin American and Caribbean Health Sciences Literature were searched for randomized controlled trials of animal protein supplementation in mothers or infants and children (≤age 5 y), evaluating measures of anthropometry (≤age 18 y). Main outcomes included birth weight, low birth weight, small for gestational age at birth; height, height-for-age, weight, weight-for-age, weight-for-length, stunting, and wasting ≤18 y of age. Data were extracted independently in duplicate, and findings pooled using inverse variance meta-analysis. Heterogeneity was explored using *I*^2^, stratified analysis, and meta-regression, and publication bias by funnel plots, Egger's test, and fill/trim methods.

**Results:**

Of 6808 unique abstracts and 357 full-text articles, 62 trials were included. The 62 trials comprised over 30,000 participants across 5 continents, including formula-based supplementation in infants and food-based supplementation in pregnancy and childhood. Maternal supplementation increased birth weight by 0.06 kg, and both formula and food-based supplementation in term infants/young children increased weight by ≤0.14 kg. Neither formula nor food-based supplementation for term infants/young children increased height, whereas the height-for-age *z*-score was increased in the food-based (+0.06 *z*-score) but not formula-based (−0.11 *z*-score) trials reporting this outcome. In term infants, the weight-for-length *z*-score was increased in trials of formula (+0.24 *z*-score) and food supplementation (+0.06 *z*-score), whereas food supplementation was also associated with reduced odds of stunting (−13%).

**Conclusions:**

Supplementation of protein from animal-source foods generally increased weight and weight-for-length in children, but with more limited effects on other growth outcomes such as attained height.

## Introduction

Suboptimal growth in young children is among the most common forms of undernutrition worldwide, with manifestations including low birth weight (LBW), low childhood height and weight, stunting, and wasting (1). With serious ramifications for physical and cognitive development, improved child growth is a major global target in the context of the United Nation's Sustainable Development Goals. For normal growth, sufficient dietary protein during pregnancy and early childhood is critical, in particular from animal-source foods due to their complete amino acid profile, contents and bioavailability of lysine, sulfur amino acids, and threonine, and associated insulin-like growth factors, iron, zinc, and vitamin B_12_ (2–6). Extreme protein deficiency leads to hypoalbuminemic malnutrition, metabolic abnormalities, and delayed development; and animal protein-rich foods and supplements have shown beneficial effects in severely undernourished children (7) and in specially formulated food supplements to treat acute malnutrition and wasting (8). However, the role of animal protein in situations of less extreme protein inadequacy (quantity or quality) is less well established.

In animal models, linear growth is sensitive to total dietary protein, for instance acting through stimulation of insulin-like growth factor-1 and its binding proteins (9–11). In humans, observational studies have found that stunting and other sub-optimal growth outcomes are often associated with diets high in staple starches and low in animal sources of protein (12–14). Yet, whereas some studies suggest that higher dietary animal protein is associated with higher growth rates in young children (5, 6), other studies suggest that increased consumption of animal protein or animal foods could result in excessive (obesogenic) growth, for example mediated by insulin-like growth factor-1 (5, 15, 16). Thus, uncertainty remains about the expected impacts of supplemental animal protein and foods in supporting optimal growth in children (13, 17–20), including effects on birth outcomes, linear growth, and prevention of stunting and wasting. In addition, such effects could vary by the period of supplementation, i.e., during pregnancy/lactation, in preterm infants, or during infancy/early childhood.

To address these important gaps in knowledge, we performed a systematic review and meta-analysis of randomized controlled trials to determine the effects of maternal, preterm infant, and term infant/child supplementation of animal protein on child birth and growth outcomes. Elucidating these relations, as well as the remaining evidence gaps, is essential to inform strategies and policymaking to reduce undernutrition globally.

## Methods

We followed the Preferred Reporting Items for Systematic Reviews and Meta-Analyses guidelines during all stages of implementation, analysis, and reporting of this meta-analysis.

### Primary exposures and outcomes

The primary exposure of interest was the consumption of protein from animal sources, including meat, seafood (including fish), dairy products (including milk), and eggs, as well as animal milk-based infant formulas, by children aged 5 y or younger and pregnant women or postpartum lactating women. The primary outcomes included birth weight and risk of LBW (<2500 g), intrauterine growth retardation, or premature birth (gestational age <37 wk); and height, height-for-age *z*-score (HAZ), weight, weight-for-age *z*-score (WAZ), weight-for-length *z*-score (WFL), and risk of stunting, assessed ≤18 y of age.

### Search strategy

Multiple electronic databases were searched for relevant articles including PubMed, EMBASE, The Cochrane Library Web of Science, Cumulative Index of Nursing and Allied Health Literature, and Latin American and Caribbean Health Sciences Literature (LILACS). This was supplemented with hand searching of citation lists and electronic searching of the first 20 “related articles” on PubMed for all included full-text publications; searches of the international standard randomized control trial number register (http://www.isrctn.com/); and contacts with experts. Searches were performed without restrictions on years or language through 25 February 2015 (and updated through to June 2018), with examples of search terms including: (dietary protein OR meat OR dietary supplements OR fortified food OR protein supplement) AND (body height OR growth child development OR pregnancy outcome OR birth weight OR stunting OR height) AND (child OR infant OR pregnant women) AND (clinical trials OR randomized controlled trial). Complete search strategies for each database are presented in the supplementary materials. Title and abstracts of all identified references were screened by 1 investigator (LP). For any potentially relevant article, the full text was retrieved and independently assessed in duplicate by 4 investigators (LP, SK, DK, VM) to determine eligibility, with discrepancies resolved by consensus.

### Study selection

#### Inclusion criteria

We included all randomized control trials that evaluated the effect of animal-source food intake in pregnancy, lactation, or children ≤age 5 y, including premature infants, low-birth-weight infants, and stunted or otherwise malnourished children, on growth outcomes as described above, including an effect measure and information to compute its standard error.

#### Exclusion criteria

We excluded studies with duration <3 mo; or where the interventions included multiple dietary or other elements that did not allow the isolation of an effect of animal protein or animal food consumption across groups. We also excluded observational studies, cross-sectional ecological studies, commentaries, general reviews, case reports, or trials conducted in populations with major chronic disease (e.g., sickle-cell disease, cystic fibrosis, HIV infection, and phenylketonuria). When duplicate publications from the same study were identified, we included the publication reporting the largest number of participants for each outcome of interest.

### Data extraction

Data from included studies were independently extracted by 4 investigators in duplicate (LP, SK, DK, VM) using a standardized electronic form, with any differences resolved by consensus. Information was extracted on the study (first author, years, design, location), participants (sample size, age, sex, race, baseline nutritional status, birth status [term, preterm], socio-economic status, baseline proportion of stunting, primary method of feeding [breastfed, formula fed]), intervention (quantity and source of animal protein, assessment methods, energy adjustment), outcome types, follow-up (duration of follow-up, dropout rate), and growth outcomes (effect size, associated measure of uncertainty). The dose of each intervention was standardized to grams of animal protein per 1000 kcal regular diet. If the precise protein content of the supplement was not reported, it was estimated using the American Diabetes Association and Academy for Nutrition and Dietetics Diabetic Exchange Lists (when not specified, dairy products were assumed to be whole milk, and meat to be medium fat) (21). When volume of formula was not reported, American Academy of Pediatrics (22) recommendations were used to estimate the amount consumed by infants at the mean age of the intervention group. If the total number of calories was not reported, this was estimated using NHANES data based on the age group of the study (23). Direct author contacts were attempted for all missing data.

### Quality assessment

Study quality was assessed using the Cochrane Collaborations risk-of-bias tool, evaluating potential for selection bias, performance bias, detection bias, attrition bias, and reporting bias through a 6-question quality control checklist (24). Each question was answered as low risk of bias (score = 1), high risk of bias (score = −1), or unclear (score = 0); and values were summed (potential range: −6 to +6) (**Supplemental Table 1**). Scores were grouped in approximate tertiles with values of −6 to 0 considered as low quality, 1–3 as medium quality, and 4–6 as high quality.

### Statistical analysis

Analyses were stratified by period of supplementation: maternal, preterm, and term/early childhood. For continuous outcomes (e.g., height, HAZ, weight, WAZ, WFL), the primary effect measure was the mean difference in changes from baseline to follow-up in the intervention compared with the control group. If mean changes from baseline were not reported, the difference in follow-up measures between treatment groups was used. For binary outcomes (e.g., risk of stunting, wasting), we extracted the reported OR across treatment groups. The SE for each effect measure was extracted or directly calculated from other reported uncertainty measures (SD, 95% CI, *P* value). We utilized the values from intent-to-treat analysis as the default. For trials reporting effects by stratum (e.g., by sex or randomized factorial design), we calculated the study-specific effect of animal protein by inverse-weighted meta-analysis across subgroups within that trial. Findings across trials were pooled using inverse variance-weighted meta-analyses (25); random effects weights were also evaluated in sensitivity analyses.

Heterogeneity was assessed using the *I*^2^ statistic, with thresholds of <25%, 50%, and >75% considered to represent low, moderate, and high heterogeneity, respectively. We evaluated prespecified sources of potential heterogeneity including country income (high, low/middle), baseline nutritional status (average/unspecified, >30% malnourished), dose of protein supplementation (> or <median), energy supplementation (isocaloric-protein, energy-protein), intervention duration (> or <median), and study quality score (low, medium/high), using stratified analyses and meta-regression to test statistical significance of potential differences. We hypothesized that benefits of supplementation would be greater in low-/middle-income countries than in high-income countries, in malnourished than in average/unspecified nutritional status, in higher than in lower dose of protein, in energy-protein supplementation than in isocaloric-protein supplementation, and in studies with longer intervention duration, and higher study quality score. In post hoc exploratory analyses, we also assessed heterogeneity by child age at baseline and at follow-up. Potential for small-study effects was evaluated by visual inspection of funnel plots and Egger's and Begg's tests (26). For both stratified analyses and evaluation of small-study effects, we focused on outcomes with at least 10 estimates to facilitate statistical power. All analyses were performed with STATA 14 (StataCorp) (2-tailed *α* = 0.05).

## Results

### Study characteristics

Of 6808 articles, 62 randomized controlled trials met eligibility criteria (**Supplemental Figure 1**, **Supplemental Information 1**), totaling 30,349 unique participants. The trials were conducted across 5 continents including 16 trials in the North America/Caribbean, 16 in Europe, 9 in Asia, 6 in Central and South America, 13 in Africa, and 2 across multiple continents (**Table 1**). Thirteen trials were conducted with pregnant women, 6 in preterm infants, and 43 in term infants/early childhood. Twenty-eight trials evaluated supplements or foods based on animal protein; 34 trials evaluated a mix of animal and plant protein. Trials of formula-based supplementation in infancy were generally isocaloric, whereas trials of food-based supplementation in pregnancy and childhood generally provided both animal protein and calories. The mean age at randomization was 31.4 y, and gestational age was on average 19.3 wk for pregnant mothers, 1 wk for preterm infants, and 9.3 mo for term infants/children. The mean intervention duration was 23.3 wk for trials during pregnancy and 26 wk for trials in infants/children; with a mean difference in protein between intervention arms of 9.85 g/1000 kcal in pregnant women, 5.6 in preterm infants, and 7.15 in term infants/children.

**TABLE 1:** Characteristics of randomized trials examining the effect of animal protein supplementation on growth outcomes in infants and children

Study author/date	Country (urban or rural)	Age, y for mothers, mo for children (GA in wk for newborns)	No. of subjects	Special population characteristics	Breast- (B) or formula-(F) fed	SES^1^	Intervention duration, wk (duration to latest follow-up, wk)	Intervention feeding	Control feeding	Difference in animal protein between groups, g/1000 kcal	Outcomes	Quality Score^4^
Pregnant mothers												
Adams 1978 (27)	USA (urban)	– (–)	145	—	—	—	19	40 g/d protein	Usual diet	18.2	BW	1
								6 g/d protein		3.2		
Adu-Afarwuah 2015 (28)	GHA (mixed)	26.7 (16.2)	708	—	—	Low	24	Lipid-based nutrient supplement with 18 micronutrients; 2.6 g/d protein	Nutrient supplement with 18 micronutrients	1.1	BW, % LBW (<2500 g), % SGA (BW <10th percentile for infants of same GA)	6
Chan 2006 (29)	USA (mixed)	16.6 (18)	49	—	—	—	22	4 servings/d of dairy (milk, yogurt, cheese); ∼32 g/d protein^2^	4 servings/d of calcium-fortified orange juice; 0 g/d protein^2^	13.9	BW	−2
Kardjati 1988 (30)	IDN (rural)	25 (–)	185	—	—	Low	13	Supplement: high-energy “jamu”; 7.1 g/d protein	Supplement: low-energy “jamu”; 6.2 g/d protein	−0.41	BW	−1
Mardones- Santander 1988 (31)	CHL (urban)	22.8 (14.5)	782	All underweight (WFL at 14 wk <95% standard)	—	Low	29	Powdered milk; 27.9 g/d protein	Powdered milk-based fortified product; 14.5 g/d protein	6.27	BW, % LBW (≤2500 g), % SGA	6
Mridha 2016 (32)	BGD (rural)	22 (13.1)	3440	31% underweight (BMI <18.5 kg/m^2^)	—	Low	27	Lipid-based nutrient supplement with iron and folic acid; 2.6 g/d protein	Nutrient supplement with iron and folic acid	1.1	BW, % LBW (<2500 g), % SGA (BW <10th percentile for infants of same GA)	6
Rush 1980 (33)	USA (urban)	– (–)	768	All underweight	—	—	15	Beverage; 10 g/d protein	Usual diet and multivitamins	2.53	BW	1
								Beverage; 6 g/d protein		16.9		
Viegas 1982 (34)	UK (urban)	28 (28)	44	—	—	Low	12	Chocolate-flavored skim milk powder, multivitamin; 10.6 g/d protein^2^	Usual diet, multivitamin, and glucose syrup^2^	4.52	BW	−1
Wohlleb 1983 (35)	TWN (rural)	26.5 (–)	110	—	—	Low	40 (92)	Chocolate-flavored, nutrient-rich supplement; 40 g/d protein	Chocolate-flavored, nutrient-rich supplement; 0 g/d protein	24.1	BW, weight, height	2
Pregnant mothers and offspring												
Ashorn 2015 (36)	MWI (rural)	Mothers: 25	1211	Neither LBW nor SGA	—	Low	23	Lipid-based nutrient supplement; 2.6 g/d protein	Nutrient supplement (same micronutrients as lipid-based nutrient supplement); 0 g/d protein	1.1	BW, weight, height, % LBW, % SGA	6
		Children: 6					52.1			2.54		
Elwood 1981 (37)	UK (urban)	Mothers: 25 (–)	—	—	—	Low	260	Milk tokens given to pregnant mothers; 19 fl oz milk average consumption; ∼18 g/d protein	Usual diet, no milk tokens	1.36	Weight, height	−2
		Children: Newborn (0 mo)	513	—	—			Milk tokens given to children up to 5 y; 19 fl oz milk average consumption; ∼18 g/d protein				
Mora 1981 (38)	COL (urban)	Mothers: 25.5 (26)	—	—	—	Low	169	Skim milk, enriched bread, vegetable oil; 38.4 g/d protein in 3rd trimester	Usual diet	18.1	BW, % LBW, weight, height	1
		Children: Newborn (0 mo)	131	—	—			Same; 30 g/d protein		19.3		
Schroeder 1995 (39)	GTM (rural)	– (–)	399	—	—	Low	13	Protein calorie supplement “Atole” (dry skim milk, sugar, incaparina); 11.5 g/d protein	Low-calorie supplement “Fresco” drink (sugar, flavorings); 0 g/d protein	4.71	BW, weight, height	1
		3	514							7.80		
		12	317							11.49		
		24	408							15.09		
		36	491							12.47		
		48	502							11.58		
		60	425							11.0		
		72	416							10.52		
Preterm infants												
Aimone 2009 (40)	CAN (mixed)	2	32	All LBW (<1800 g) and some SGA^3^	Mainly B	—	12 (44)	Nutrient-enriched human milk; 3.6 g protein/100 mL human milk	Unfortified human milk	9.04	Weight, height	2
Amesz 2010 (41)	NL (urban)	—	93	Some LBW (≤1750 g)	F	—	24 (34)	Nutrient-enriched formula; 1.7 g protein/100 mL^2^	Standard term formula; 1.47 g protein/100 mL^2^	3.43	WAZ, HAZ	−2
Carver 2001 (42)	USA (urban)	0	54	All LBW (BW ≤1800 g)	F	—	60	Postdischarge formula; 2.6 g/100 kcal	Term formula; 2.1 g/100 kcal	4.01	Weight, height	5
Cooke 2010 (43)	UK (mixed)	0	113	All LBW (≤1500 g)	F	—	26 (52)	Preterm formula to 6 mo corrected age; 2.2 g protein/100 mL^2^	${\rm T}$erm formula to 6 mo corrected age; 1.4 g protein/100 mL^2^	8.79	Weight	−2
Embleton 2005 (44)	UK (urban)	0.75	50	All LBW (≤1750 g) and SGA (SDS < −2 SD at that gestation)	F	High	18	Formula; 2.6 g protein/100 mL^2^	Formula; 2.2 g protein/100 mL^2^	4.98	Weight, height, WFL, HAZ	−2
								Formula; 2.4 g protein/100 mL^2^		2.48		
Koo 2006 (45)	USA (urban)	0	76	All LBW (630–1620 g) and SGA^3^	F	—	52	Nutrient-enriched formula; 2.6 g protein/100 kcal	Term formula; 2.14 g protein/100 kcal	6.79	Weight, height, WAZ, HAZ	6
Term infants (formula intervention)												
Borschel 2013 (46)	USA (mixed)	0.19	157	Neither LBW nor SGA	F	—	15	Formula; 2.03 g protein/100 mL^2^	Formula; 1.89 g protein/100 mL^2^	2.07	Weight, height	−2
Fazzolari-Nesci 1992 (47)	SWE (mixed)	0	10	Neither LBW nor SGA	F	—	12	Formula; 0.157 g protein/mL^2^	Formula; 0.137 g protein/mL^2^	1.6	Weight, height	2
Fleddermann 2014 (48)	SRB	0.93	164	Neither LBW nor SGA	F	—	13.1	Formula; 1.5 g protein/100 mL^2^	Formula; 1.3 g protein/100 mL	2.79	Weight, height	6
Fomon 1995 (49)	USA (urban)	0	29	Neither LBW nor SGA	F	High	15	Formula; 1.5 g protein/100 mL^2^	Formula; 0.83 g protein/100 mL^2^	8.08	Weight, height	−1
Graham 1996 (50)	PER (urban)	9.8	30	All stunted (>3rd percentile NCHS WFL), all SGA^3^	F	Low	13	Formula: 6.7%E protein, 8.0%E protein	Formula: 5.5%E protein	5.16, 9.27	Weight, height	5
		19.6	28					Formula: 6.4%E protein , 8.0%E protein	Formula: 4.7%E protein	3.98, 8.93		
Hanning 1992 (51)	CAN (mixed)	0	95	Neither LBW nor SGA	F	—	12	Formula; 1.59 g protein/100 mL^2^	Formula; 1.33 g protein/100 mL^2^	3.89	Weight, height	3
Koletzko 2009 (15)	BEL, GER, ITA, POL, ES (urban)	0.5	636	—	F	Medium	49.5 (101)	Formula; 2.2 g protein/100 kcal followed by 4.4 g protein/100 kcal	Formula; 1.77 g protein/100 kcal followed by 2.9 g protein/100 kcal	16.65	Weight, height, WAZ, HAZ, WFL	6
Larnkjaer 2009 (52)	DNK (urban)	9.1	86	Neither LBW nor SGA	Mainly F	High	13	Whole milk; ∼10 g/d protein	Formula; ∼1.35 g protein/100 mL	29.6	Weight, height,	6
Lien 2004 (53)	USA (mixed)	0.25	135	Neither LBW nor SGA	F	—	12	Formula; 1.5 g protein/100 mL	Formula; 1.44 g protein/100 mL	0.57	Weight, height	5
Lonnerdal 1990 (54)	TWN (urban)	0	—	—	Mainly F	—	12	Formula; 1.4 g protein/100 mL^2^	Formula; 1.29 g protein/100 mL^2^	1.94	Weight, height	6
						—		Formula; 1.5 g protein/100 mL^2^		3.88		
Lonnerdal 1998 (55)	SWE (mixed)	0	22	Neither LBW nor SGA	F	—	19.8	Formula; 1.5 g protein/100 mL	Formula; 1.3 g protein/100 mL	1.6	Weight, height	−3
Oropeza-Ceja 2018 (56)	MEX (urban)	0.7	96	—	F	Medium	13.9	Formula; 1.9 g protein/100 kcal	Formula; 1.43 g protein/100 kcal	4.7	Weight, height, WAZ, HAZ, WFL	6
								Formula; 2.18 g protein/100 kcal		7.5		
Raiha 2002 (57)	ITA (urban)	0^5^	58	Neither LBW nor SGA	F	Medium	13	Formula; 2.2 g protein/100 kcal^2^	Formula; 1.8 g protein/100 kcal^2^	0.46	Weight, height, WAZ, HAZ	1
Rzehak 2009 (58)	GER (urban)	0	235	Neither LBW nor SGA	F	High	16 (256)	Formula; 1.6 g protein/100 mL^2^	Formula; 1.4 g protein/100 kcal	2.67	Weight, height	−1
								Formula; 1.9 g protein/100 mL^2^		6.72		
Schmelzle 2003 (59)	GER (mixed)	0.23	101	Neither LBW nor SGA	F	—	11	Formula; 1.7g protein/100 mL	Formula; 1.5g protein/100 mL	4.02	Weight, height	4
Timby 2014 (60)	SWE (urban)	1.5	148	Neither LBW nor SGA	F	High	26 (52)	Formula; 1.27 g protein/100 mL	Formula; 1.2 g protein/100 mL	−0.46	WAZ, HAZ	−1
Turck 2006 (61)	FRA (urban)	0.12	74	Neither LBW nor SGA	F	High	17	Formula; 2.6 g protein/100 kcal^2^	Formula; 1.8 g protein/100 kcal^2^	9.45	Weight, height	−1
Weber 2014 (62)	BEL, GER, ITA, POL, ES (mixed)	0.46	448	Neither LBW nor SGA	F	Medium	52 (312)	Formula; ∼2.6 g protein/100 mL^2^	Formula; ∼1.4 g protein/100 mL^2^	22.8	Weight, height, WAZ, HAZ	−1
Ziegler 2003 (63)	USA (urban)	0.25	33	Neither LBW nor SGA	F	High	15	Formula; 2.39 g protein/100 mL^2^	Formula; 1.92 g protein/100 mL^2^	5.35	Weight, height	1
Ziegler 2015 (64)	USA (urban)	3	174	—	F	High	38.7	Formula; 1.39 g protein/100 mL	Formula; 1.08 g protein/100 mL	5.4	WAZ, HAZ	5
Term infants/children (whole-food intervention)												
Ackatia-Armah 2015 (65)	MLI (rural)	14.6	623	All stunted (WFL < −2 SD)	—	Low	12	Corn–soy blend “plus plus”: refined cereal–legume–milk blend; 18.4 g/d protein^2^	Less refined cereal–legume flour mix: millet, beans, sugar, and oil; 15.5 g/d protein^2^	2.19	Weight, length, WFL	3
Alarcon 2003 (66)	TWN, PHL (mixed)	48.5	91	All stunted (<25th percentile WFL)	Mainly B	—	12	Pediasure, 40 mL/kg/d; 12%E as protein	Usual diet	38.2	Weight, height, WAZ, HAZ	−2
Bauserman 2015 (67)	DRC (rural)	6	222	34.7% stunted (HAZ < −2 SD)	B	Low	52	Caterpillar cereal; 6.9 g/d protein for 6–12 mo of age and 10.3 g/d protein for 12–18 mo of age	Usual diet	8.5	WAZ, HAZ, WFL, stunting, wasting	6
Christian 2015 (68)	BGD (rural)	6.2	2962	−24.7% stunted (HAZ < −2 SD)	—	Low	52	Chickpea-based ready-to-eat food containing sugar, soybean oil, and whole-milk powder; 15 g protein/100 g	Usual diet	4	Weight, height, HAZ, HAZ%, WFL%	0
								Lentil-based ready-to-eat food containing sugar, soybean oil, and whole-milk powder; 11 g protein/100 g		5.47		
Fabiansen 2017 (69)	BFA (rural)	11.5	1609	All with moderate acute malnutrition (MUAC ≥ 115 mm and <125 mm and/or WFL ≥±3 and <±2)	—	Low	12	Corn–soy blend or lipid-based supplement with dehulled or isolate soy (factorial trial design); 20% of total protein from skimmed milk	Corn–soy blend or lipid-based supplement with dehulled or isolate soy (factorial trial design); no skimmed milk	2.79	Weight, height, WFL	5
								Corn–soy blend or lipid-based supplement with dehulled or isolate soy (factorial trial design); 50% of total protein from skimmed milk		6.99		
He 2005 (70)	CHN (urban)	50.6	402	All stunted^3^	—	Medium	39	1 cup yogurt/d (125 g); 8 g/d protein	Usual diet	4.35	Weight, height, WAZ, HAZ	4
Heikens 1993 (71)	JAM (urban)	14.6	75	All underweight (<80% NCHS WAZ) and malnourished^3^	—	Low	13 (26)	High-energy supplement: full-milk cream powder, sugar, oil mixed with water; 20.6 g/d protein	Usual diet	15.62	Weight, height	0
Heikens 1989 (72)	JAM (urban)	14.4	82	All underweight (<80% NCHS WAZ) and malnourished^3^	—	Low	13 (26)	High-energy supplement: full-milk cream powder, sugar, oil mixed with water; 20.6 g/d protein	Usual diet	15.62	Weight, height	0
Iannotti 2017 (73)	ECU (rural)	7.6	163	38% stunted (HAZ < −2 SD)	—	Low	25.8	1 egg; 6.46 g/d protein	Usual diet	6.46	WAZ, HAZ, WFL, stunting	4
Krebs 2012 (74)	GTM, DRC, ZMB, PAK (mixed)	6	1062	33% stunted (HAZ < −2 SD), some SGA^3^	Mixed	Low	52	Lyophilized beef; ∼8 g/d protein^2^	Precooked rice and soy flour with micronutrients; ∼2 g/d protein^2^	6.26	WAZ, HAZ, WFL, HAZ%, height	6
Lin 2008 (75)	MWI (rural)	6	240	—	B	Low	52	Corn porridge fortified with fish powder; 11.2 g/d protein^2^	Peanut and soy-based fortified spread; 5.8 g/d protein^2^	4.69	Weight, height	1
Long 2012 (76)	KEN (rural)	25.5	193	27% stunted^3^	—	Low	22	Meat porridge; 13 g/d protein^2^	Plain millet-based porridge (sugar, margarine); 3.4 g/d protein^2^	5.77	Weight, height, WAZ, WFL, HAZ	6
								Whole-milk porridge; 5.9 g/d protein^2^		1.41		
Maleta 2015 (77)	MWI (rural)	5.9	1291	29.3% moderately to severely stunted (HAZ < −2.0)	Mainly F	Low	52	Milk containing maize-based porridge; 2.5 g/d protein	Nonmilk containing maize-based porridge; 1.0 g/d protein	1.5	Weight, height, WAZ, HAZ, WFL, stunting, wasting	4
								Milk containing maize-based porridge; 5.0 g/d protein	Milk containing maize-based porridge; 2.0 g/d protein	3.0		
Mangani 2015 (78)	MWI (rural)	6	376	8.5% stunted (HAZ < −3 SD)	—	Low	52	Milk-protein–based fortified soy–corn flour; 8.2 g/d protein	Usual diet	6.49	Weight, height, WAZ, WFL, HAZ	6
Nikiema 2014 (79)	BFA (rural)	13.2	1369	All stunted (WFL < −2 SD)	—	Low	12	Corn–soy blend with added micronutrients: maize, soya, sugar, dried skim milk, and soybean oil; 10.4 g/d protein^2^	Ready-to-use supplementary food: peanut butter, vegetable oil, sugar, soy flour, shea butter, added micronutrients; 8.7 g/d protein^2^	1.47	Height (length gain), WFL, HAZ	1
Schlossman 2017 (80)	GNB (rural)	15.7	327	All with mild to moderate acute malnutrition (WAZ <1.0, or HAZ < 2.0, or WFL < 2.0)	—	Low	12.9	Soy- and dairy-protein paste; 0.6 g/d protein	Usual diet	0.773	WAZ, HAZ, WFL	3
		38.8	159					Soy- and dairy-protein paste; 1.2 g/d protein		1.2		
Simondon 1996 (81)	COG, SEN, BOL, NCL (mixed)	4	447	Neither LBW nor SGA	Mixed	Medium	12	Precooked wheat, maize, soybean flour, milk powder, soybean oil, palm oil, sugar; 6.74 g/d protein	Usual diet	6.47	Weight, height	3
Skau 2015 (82)	KHM (rural)	5.9	180	18% stunted (HAZ < −2 SD)	—	Low	38.7	Vegetable oil, maize, soya, skimmed milk powder; 16.8 g/d protein	Vegetable oil, sugar, maize, soya; 14.6 g/d protein	1.76	Weight, height, WAZ, WFL, HAZ	4
Stobaugh 2016 (7)	MWI, MOZ (rural)	16.4	2230	All with moderate acute malnutrition (MUAC ≥ 115 mm and <125 mm without bipedal edema)	Mainly B	Low	12	Dairy-based ready-to-use supplementary food	Soy-based ready-to-use supplementary food		Weight, height, WFL	6
Tang 2014 (83)	USA (urban)	5	42	Neither LBW nor SGA	B	—	17	Pureed meat and gravy; ∼8 g/d protein	Cereal; ∼2–3 g/d protein	30.3	WAZ, WFL, HAZ	−1
Tang 2014 (84)	CHN (rural)	7	1318	30% stunted^3^, neither LBW nor SGA	Mixed	Low	52	Boiled pork, every other day; ∼14.8 g/d protein^2^	Commercially available packaged pressed rice cereal; ∼3.9 g/d protein^2^	0.88	Weight, height, WAZ, WFL, HAZ	−1
Tavill 1969 (85)	MAR (urban)	6	88	Neither LBW nor SGA	—	Low	26	Fish-protein concentrate; ∼13 g/d protein	Usual diet	12.97	Weight, height	−1
Walker 1996 (86)	JAM (urban)	18.7	63	All stunted (HAZ < −2 SD)	—	—	52 (103.2)	Milk-based formula, skim milk powder, and cornmeal; 14 g protein/100 mL	Usual diet	15.2	Weight, height, WFL, HAZ	−1

1 If not directly reported, estimated from the study descriptorsby 2 investigators independently and in duplicate. BEL, Belgium; BFA, Burkina Faso; BGD, Bangladesh; BOL, Bolivia; BW, birth weight; CAN, Canada; CHL, Chile; CHN, China; COG, Congo; COL, Colombia; DNK, Denmark; DRC, Democratic Republic of Congo; ECU, Ecuador; ES, Spain; FRA, France; GA, Gestational age; GER, Germany; GHA, Ghana; GNB, Guinea-Bissau; GTM, Guatemala; HAZ, height-for-age *z*-score; HAZ%, percentage height-for-age *z*-score(stunting); IDN, Indonesia; ITA, Italy; JAM, Jamaica; KEN, Kenya; KHM, Cambodia; LBW, low birth weight; MAR, Morocco; MEX, Mexico; MLI, Mali; MOZ, Mozambique; MUAC, midupper arm circumference; MWI, Malawi; NCHS, National Center for Health Statistics; NCL, New Caledonia; NL, Netherlands; PAK, Pakistan; PER, Peru; PHL, Philippines; POL, Poland; SEN, Senegal; SES, socio-economic status; SGA, small for gestational age; SRB, Serbia; SWE, Sweden; TWN, Taiwan; UK, United Kingdom; USA, United States of America; WAZ, weight-for-age *z*-score; WFL, weight-for-length *z*-score; WFL%, percentage weight-for-length *z*-score; ZMB, Zambia; –, information not applicable or not available.

2 The intervention and control supplements were isocaloric.

3 Not defined in the report.

4 The Cochrane Collaboration's tool for assessing risk of bias was used to assess potential for selection bias, performance bias, detection bias, attrition bias, and reporting bias through a 6-question quality control checklist (24). Each question was answered as low risk of bias (score = 1), high risk of bias (score = −1), or unclear (score = 0); and values were summed (potential range: −6 to +6). Scores were grouped in approximate tertiles with values of −6 to 0 considered as low quality, 1–3 as medium quality, and 4–6 as high quality.

5 Population is <25% or >75% males.

### Maternal supplementation

**FIGURE 1:**
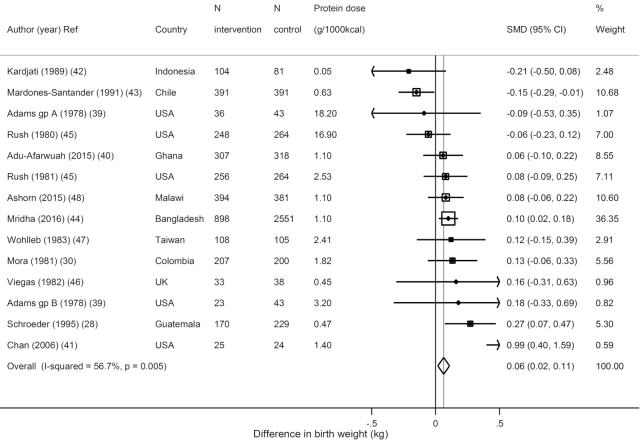
Effects of protein supplementation on birth weight in kilograms from 14 estimates in 12 trials including 8132 subjects. SMD, standardized (weighted) mean difference.

Among trials during pregnancy, protein supplementation significantly increased birth weight (*N* = 14 estimates from 12 trials, *n* = 8132 total participants; weighted mean difference [WMD] = 0.06 kg; 95% CI: 0.02, 0.11 kg; *I*^2^ = 56.7%) (**Figure 1**). Maternal supplementation did not significantly reduce the risk of LBW (*N* = 5, *n* = 6121; OR: 0.89; 95% CI: 0.78, 1.02; *I*^2^ = 0.8%) or small for gestational age (SGA) (*N* = 4, *n* = 5674; OR: 0.98; 95% CI: 0.87, 1.10; *I*^2^ = 80.3%) (**Supplemental Figure 2**), or increase height (*N* = 3, *n* = 1490; WMD = 0.01 cm; 95% CI: −0.09, 0.11 cm; *I*^2^ = 59.7%) or weight (*N* = 2, *n* = 636; WMD = −0.08 kg; 95% CI: −0.23, 0.08 kg; *I*^2^ = 0.0%) during later childhood (**Supplemental Figure 3**).

Three trials supplemented both mothers (during pregnancy and/or breastfeeding) and their children after birth, all with combined energy–animal protein supplementation: increases were seen in both child height (*N* = 2, *n* = 3276; WMD = 0.09 cm; 95% CI: 0.02, 0.15 cm; *I*^2^ = 61.8%) and weight (*N* = 3, *n* = 4227; WMD = 0.10 kg; 95% CI: 0.04, 0.16 kg; *I*^2^ = 71.6%) (**Supplemental Figure 4**).

### Preterm infant supplementation

Supplementation of preterm infants (comparing formula with higher compared with lower animal protein) did not significantly affect child height (*N* = 5, *n* = 262; WMD = 0.06 cm; 95% CI: −0.22, 0.34 cm; *I*^2^ = 96.9%) and reduced HAZ (*N* = 4, *n* = 269; WMD = −1.31 *z*-score; 95% CI: −1.60, −1.01; *I*^2^ = 96.8%) (**Supplemental Figure 5**), although these trials were quite small. Preterm supplementation did not significantly affect child weight (*N* = 6, *n* = 373; WMD = 0.19 kg; 95% CI: −0.03, 0.42 kg; *I*^2^ = 96.2%) (**Supplemental Figure 6**). Only 2 trials reported weight for age, which decreased (*N* = 2, *n* = 169; WMD = −0.81 *z*-score; 95% CI:−1.16, −0.46; *I*^2^ = 98.4%), and 1 trial provided 2 estimates for weight for length, which also decreased (*N* = 2, *n* = 100; WMD = −1.57 *z*-score; 95% CI: −2.02, −1.12; *I*^2^ = 0.0%) (**Supplemental Figure 7**).

### Term infant/child supplementation

**FIGURE 2:**
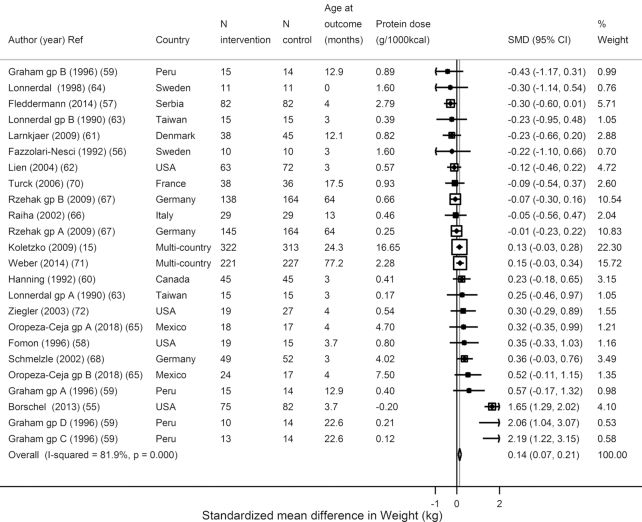
Effects of term child protein formula supplementation on weight in kilograms from 24 estimates from 18 trials including 2923 subjects. SMD, standardized (weighted) mean difference.

**FIGURE 3:**
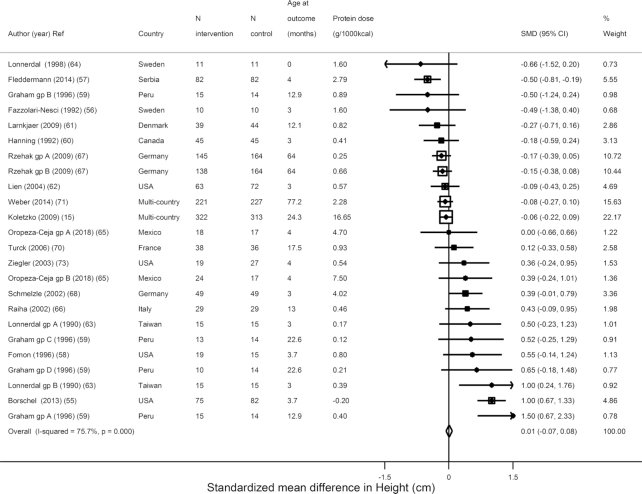
Effects of term child protein formula supplementation on height in centimeters from 24 estimates in 18 trials including 2920 subjects. SMD, standardized (weighted) mean difference.

In trials of formula supplementation (higher compared with lower protein content) among term infants/children, supplementation significantly increased weight (*N* = 24, *n* = 2923; WMD = 0.14 kg; 95% CI: 0.07, 0.21 kg; *I*^2^ = 81.9%) (**Figure 2**) but not height (*N* = 24, *n* = 2920; WMD = 0.01 cm; 95% CI: −0.07, 0.08 cm; *I*^2^ = 75.7%) (**Figure 3**). Only 7 estimates from 6 trials reported weight- and height-for-age *z*-scores: WAZ was not reduced (*N* = 7, *n* = 1532; WMD = −0.01 *z*-score; 95% CI: −0.11, 0.09; *I*^2^ = 95.1%), but HAZ (*N* = 7, *n* = 1532, WMD = −0.11 *z*-score; 95% CI: −0.22, −0.0.01; *I*^2^ = 90.3%) was reduced (**Supplemental Figure 8**). Only 3 studies reported WFL: WFL was increased (*N* = 3, *n* = 711; WMD = 0.24 *z*-score; 95% CI: 0.09, 0.38; *I*^2^ = 0.0%) (**Supplemental Figure 9**).

**FIGURE 4:**
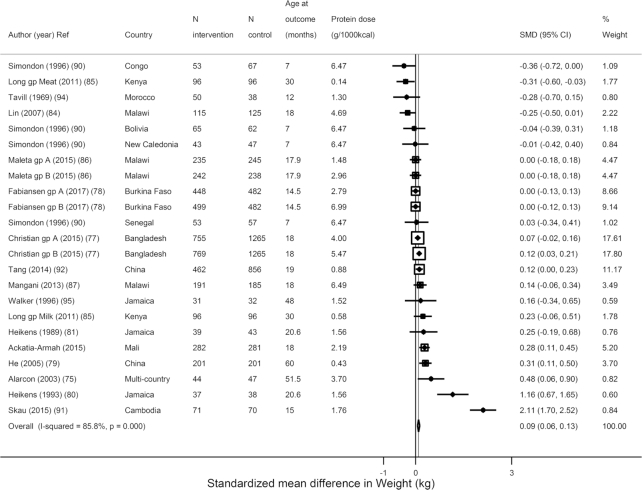
Effects of term child protein food-based supplementation on weight in kilograms from 23 estimates from 16 trials including 11,195 subjects. SMD, standardized (weighted) mean difference.

**FIGURE 5:**
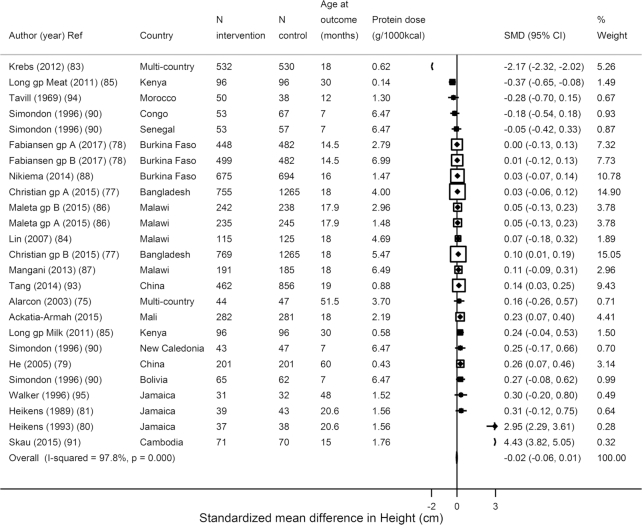
Effects of term child protein food-based supplementation on height in centimeters from 25 estimates from 18 trials including 13,626 subjects. SMD, standardized (weighted) mean difference.

Similar to formula trials, the trials testing food-based animal protein showed that supplementation significantly increased weight (*N* = 23 estimates, *n* = 11,195; WMD: 0.09 kg; 95% CI: 0.06, 0.13 kg; *I*^2^ = 85.8%) (**Figure 4**) but not height (*N* = 25, *n* = 13,626; WMD = −0.02 cm; 95% CI: −0.06, 0.01 cm; *I*^2^ = 97.8%) (**Figure 5**). However, in 19 trials assessing *z*-scores, HAZ increased (*N* = 19, *n* = 11,098; WMD = 0.06 *z*-score; 95% CI: 0.02, 0.10; *I*^2^ = 57.9%) (**Supplemental Figure 10**). In these food-based trials, the source was most often milk; yogurt, fish, and red meat were also used. Consistent with the overall weight effects, a tendency toward an increase was seen in weight for age (*N* = 15, *n* = 5611; WMD = 0.05 *z*-score; 95% CI: 0.00, 0.10; *I*^2^ = 80.6%) and an increase in weight for length (*N* = 19, *n* = 11,251; WMD = 0.06 *z*-score; 95% CI: 0.02, 0.10; *I*^2^ = 87.2%) (**Supplemental Figure 11**). Only 3 trials evaluated stunting, finding a significant reduced risk of stunting (*N* = 6 estimates, *n* = 5138; OR: 0.87; 95% CI: 0.77, 0.97; *I*^2^ = 0), but no reduced risk of wasting (*N* = 5 estimates, *n* = 5267; OR: 0.99; 95% CI: 0.84, 1.16; *I*^2^ = 0.0%) (**Supplemental Figure 12**).

### Potential sources of heterogeneity

Using stratified analyses and meta-regression, we evaluated prespecified potential sources of heterogeneity (**Table 2**). Among trials of pregnant women evaluating birth weight, no significant heterogeneity was identified by country income, baseline nutritional status, dose of protein supplementation, intervention duration, or study quality score. Compared with isocaloric-protein supplementation (one trial only), energy-protein supplementation to pregnant women was more effective in increasing birth weight (*P* heterogeneity = 0.002; birth weight WMD = 0.089 kg; 95% CI: 0.040, 0.137 kg).

**TABLE 2:** Sources of potential heterogeneity among randomized trials examining the effect of animal protein supplementation on growth outcomes in infants and children^1^

	Pregnant mothers		Term children, formula-based				Term children, food-based									
	Birth weight, kg		Height, cm		Weight, kg		Height, cm		Weight, kg		Height-for-age, *z*-score		Weight-for-age, *z*-score		Weight-for-length, *z*-score	
Potential sources of heterogeneity	*N*	Pooled estimate (95% CI)	*N*	Pooled estimate (95% CI)	*N*	Pooled estimate (95% CI)	*N*	Pooled estimate (95% CI)	*N*	Pooled estimate (95% CI)	*N*	Pooled estimate (95% CI)	*N*	Pooled estimate (95% CI)	*N*	Pooled estimate (95% CI)
Country income																
High	7	0.064 (−0.037, 0.166)	18	0.111 (0.036, 0.187)	18	−0.016 (−0.092, 0.059)	0		0		1	−0.075 (−0.717, 0.566)	1	0.097 (−0.545, 0.739)	1	0.185 (−0.458, 0.828)
Low/middle	7	0.063 (0.012, 0.115)	6	0.632 (0.323, 0.942)	6	0.361 (0.063, 0.660)	25	−0.024 (−0.059, 0.010)	23	0.095 (0.057, 0.133)	18	0.059 (0.021, 0.097)	15	0.050 (−0.004, 0.103)	18	0.060 (0.023, 0.098)
*P* heterogeneity, univariate (multivariate)^2^		0.986		0.001* (0.621)		0.016* (0.680)		NA		NA		0.682		0.886		0.705
Baseline nutritional status^3^																
Average/unspecified	10	0.107 (0.033, 0.180)	20	0.120 (0.045, 0.194)	20	−0.010 (−0.085, 0.064)	14	0.082 (0.033, 0.132)	14	0.071 (0.022, 0.120)	9	0.056 (0.004, 0.108)	7	−0.031 (−0.121, 0.058)	11	0.161 (0.071, 0.252)
>30% malnourished	4	0.036 (−0.023, 0.094)	4	0.805 (0.386, 1.225)	4	0.480 (0.084, 0.875)	11	−0.130 (−0.179, −0.081)	9	0.131 (0.071, 0.190)	10	0.061 (0.006, 0.117)	8	0.094 (0.028, 0.160)	12	0.040 (−0.001, 0.081)
*P* heterogeneity, univariate (multivariate)^2^		0.140		0.002* (0.341)		0.017* (0.539)		<0.001* (0.578)		0.128		0.891		0.026* (0.671)		0.017* (0.595)
Dose of protein supplementation^4^																
>Median	7	0.058 (−0.030, 0.147)	8	0.108 (0.006, 0.211)	8	−0.088 (−0.191, 0.015)	16	0.102 (0.059, 0.146)	16	0.097 (0.053, 0.140)	9	0.100 (0.046, 0.154)	8	0.082 (−0.007, 0.171)	10	0.131 (0.081, 0.182)
<Median	7	0.065 (0.012, 0.119)	16	0.175 (0.069, 0.280)	16	0.104 (−0.000, 0.209)	9	−0.245 (−0.302, −0.187)	7	0.089 (0.012, 0.166)	10	0.019 (−0.034, 0.072)	7	0.032 (−0.034, 0.098)	9	−0.024 (−0.079, 0.032)
*P* heterogeneity, univariate (multivariate)^2^		0.894		0.376		0.010* (0.224)		0.001* (0.189)		0.864		0.037* (0.275)		0.382		0.001* (0.163)
Energy supplementation^5^																
Isocaloric-protein	1	−0.150 (−0.290, −0.009)	24	0.141 (0.067, 0.214)	24	0.007 (−0.067, 0.080)	4	0.058 (−0.018, 0.133)	4	0.038 (−0.038, 0.113)	2	0.128 (−0.050, 0.306)	2	0.127 (−0.051, 0.306)	5	0.054 (−0.018, 0.126)
Energy-protein	13	0.089 (0.040, 0.137)	0		0		21	−0.047 (−0.086, −0.007)	19	0.114 (0.071, 0.158)	17	0.055 (0.017, 0.094)	13	0.042 (−0.013, 0.098)	14	0.063 (0.020, 0.107)
*P* heterogeneity, univariate (multivariate)^2^		0.002*		NA		NA		0.016* (0.772)		0.084		0.435		0.373		0.836
Intervention duration^4^																
>Median	5	0.056 (−0.001, 0.114)	6	0.064 −(0.029, 0.157)	6	−0.101 (−0.194, −0.008)	14	−0.081 (−0.125, −0.038)	13	0.101 (0.056, 0.146)	15	0.066 (0.024, 0.108)	15	0.023 (−0.033, 0.079)	12	0.057 (−0.001, 0.116)
<Median	9	0.076 (−0.173, 0.284)	18	0.270 (0.150, 0.391)	18	0.184 (0.064, 0.303)	11	0.080 (0.021, 0.138)	10	0.080 (0.011, 0.150)	4	0.025 (−0.064, 0.114)	3	0.285 (0.119, 0.451	7	0.063 (0.015, 0.112)
*P* heterogeneity, univariate (multivariate)^2^		0.694		0.008* (0.507)		0.001* (0.954)		<0.001* (0.483)		0.627		0.417		0.003* (0.205)		0.882
Study quality score^6^																
Low (−6 to 0)	3	0.055 (0.017, 0.109)	7	0.182 (0.073, 0.290)	7	0.013 (−0.095, 0.121)	8	0.105 (0.051, 0.159)	8	0.114 (0.060, 0.167)	6	0.107 (0.052, 0.161)	3	0.198 (0.090, 0.306)	3	0.040 (−0.069, 0.148)
Medium/high (1 to 6)	11	0.064 (0.017, 0.111)	17	0.106 (0.006, 0.206)	17	0.001 (−0.099, 0.101)	17	−0.119 (−0.165, −0.073)	15	0.076 (0.022, 0.130)	13	0.013 (−0.040, 0.066)	12	0.003 (−0.058, 0.064)	16	0.064 (0.024, 0.103)
*P* heterogeneity, univariate (multivariate)^2^		0.944		0.316		0.867		<0.001* (0.685)		0.327		0.015* (0.419)		0.002* (0.103)		0.687
Age at supplementation^4^,																
>Median	5	0.043 (−0.029, 0.115)	5	0.304 (0.003, 0.605)	5	0.138 (−0.154, 0.430)	18	−0.050 (−0.087, −0.013)	16	0.095 (0.054, 0.135)	15	0.058 (0.018, 0.098)	11	0.061 (0.001, 0.121)	15	0.068 (0.029, 0.108)
<Median	9	0.068 (−0.031, 0.167)	19	0.130 (0.055, 0.206)	19	−0.002 (−0.078, 0.073)	7	0.177 (0.074, 0.280)	10	0.098 (−0.004, 0.199)	7	0.064 (−0.050, 0.179)	4	0.009 (−0.106, 0.123)	4	−0.004 (−0.118, 0.111)
*P* heterogeneity, univariate (multivariate)^2^		0.976		0.273		0.363		0.001* (0.395)		0.958		0.920		0.424		0.243
Age at follow-up^4^,																
>Median	7	0.061 (−0.027, 0.150)	10	0.098 (0.008, 0.189)	10	−0.087 (−0.177, 0.004)	12	0.092 (0.037, 0.146)	12	0.077 (0.023, 0.132)	12	0.105 (0.042, 0.168)	14	0.086 (0.023, 0.150)	16	0.031 (−0.014, 0.076)
<Median	7	0.051 (−0.002, 0.105)	14	0.224 (0.097, 0.351)	14	0.187 (0.062, 0.313)	13	−0.103 (−0.148, −0.058)	11	0.111 (0.059, 0.164)	7	0.032 (−0.015, 0.079)	4	−0.034 (−0.130, 0.063)	6	0.124 (0.058, 0.190)
*P* heterogeneity, univariate (multivariate)^2^		0.852		0.113		0.001* (0.282)		<0.001* (0.605)		0.378		0.068		0.042* (0.720)		0.023* (0.125)

1 Only pooled analyses with 10 or more estimates were evaluated in these stratified analyses and tests for heterogeneity. For each stratum, the pooled estimate (95% CI) was calculated using inverse variance-weighted meta-analyses. *Values indicate *P* < 0.05. HAZ, height-for-age *z*-score.

2 *P* value for heterogeneity calculated using univariate meta-regression analysis. For each outcome, multivariate meta-regression was subsequently performed including all factors with univariate *P* < 0.05 together in the same model.

3 Defined according to the study definition (e.g., HAZ < 2SD).

4 Median value defined within each target population separately (e.g., maternal supplementation, term infants).

5 When energy intakes were not specified, an intervention compared with a “usual diet” control was considered to be an energy-protein (not isocaloric-protein) intervention, whereas a control that used a specific control supplement was considered to be isocaloric-protein.

6 Study quality was assessed using the Cochrane Collaboration risk-of-bias tool, evaluating potential for selection bias, performance bias, detection bias, attrition bias, and reporting bias through a 6-question quality control checklist (24). Each question was answered as a low risk of bias (score = 1), high risk of bias (score = −1), or unclear (score = 0), and values were summed (potential range: −6 to +6). Scores were grouped in approximate tertiles with values of −6 to 0 considered as low quality, 1–3 as medium quality, and 4–6 as high quality.

For trials of formula-based supplementation in term children, univariate meta-regression suggested that studies from low- and middle-income countries (*N* = 6) showed larger increases in both weight (*P* heterogeneity = 0.001) and height (*P* heterogeneity = 0.02) than studies from high-income countries (*N* = 18); similar stratification was identified by baseline nutritional status and intervention duration. Doses of animal protein supplementation below median showed larger effects. No significant heterogeneity was identified by study quality score or in multivariate meta-regression.

Among trials of food-based supplementation in term children, effects on height were smaller, in studies of malnourished compared with average/unspecified nutritional status (*P* heterogeneity < 0.001), whereas effects on WAZ and WFL were greater in studies of average/unspecified nutritional status (*P* heterogeneity < 0.026 for both). In univariate meta-regression, potential heterogeneity was also identified by dose of protein supplementation, presence of energy supplementation, intervention duration, and study quality score for studies of the effect of height. Heterogeneity was identified by dose and study quality score in studies of HAZ meta-regression, and for baseline nutritional status, intervention duration and study quality, for studies of the effect on WAZ. Only baseline nutritional status and animal protein supplementation dose shower heterogeneity effects in studies of WFL. None of these interactions remained significant in multivariate meta-regression. No variables were found to drive heterogeneity in studies of the effect on weight.

In post hoc analyses of interaction by age at follow-up, children below the median age at follow-up showed stronger effects in the association of formula-based supplementation and weight and the association of food-based supplementation and WFL. Above the median age at follow-up was associated with stronger effects of term children food-based supplementation on height and WAZ.

### Evaluation of small-study effects

Visual inspection of funnel plots and Egger's and Begg's tests did not provide any evidence for meaningful small-study effects (**Supplemental Figure 13**).

## Discussion

This systematic review and meta-analysis of 62 controlled trials comprising over 30,000 participants across 5 continents found that supplementation of protein from animal-source foods generally increased weight in children; yet, it had limited effects on other outcomes such as attained height or stunting. For instance, maternal supplementation increased birth weight by 0.06 kg (with no significant effect on LBW or SGA), and both formula and food-based supplementation in term infants/young children increased weight by 0.14 and 0.09 kg, respectively. The strongest effects were seen in trials where both mothers and children were supplemented, with 100-g increases in weight and a mean 0.1-cm increase in height. However, neither formula nor food-based supplementation for term infants/young children increased height, whereas HAZ was increased in food-based (+0.06 *z*-score) but not formula-based trials (−0.11 *z*-score) reporting this outcome. Whether this latter difference is due to chance or benefits of nonprotein components of animal-source foods remains unclear, and the limited number of studies reporting on HAZ outcomes after term formula supplementation (*N* = 7) adds uncertainty about this finding. We conducted a post hoc meta-analysis of height and weight in studies of food-based supplementation in term children in the subgroup of studies that reported HAZ and WAZ, respectively, as an outcome. However, we still did not find a positive effect of food-based supplementation on height, and the significant effect on weight was maintained in this subgroup of studies. Supplementation in preterm infants did not significantly improve growth outcomes; in contrast, lower height for age, weight for age, and weight for length (although based on only 6 trials totaling <400 participants) were shown. In sum, these findings do not provide strong evidence for the benefits of animal-protein supplementation in mothers and preterm infants, and during infancy/early childhood on growth outcomes other than weight and WFL, and stunting for food supplementation in term children; the potential effects of food-based supplementation on HAZ require further study.

Formula-based trials generally provided isocaloric animal protein supplementation, whereas trials in pregnant mothers and food-based trials in children generally provided both animal protein and additional calories from foods (“balanced energy–animal protein supplementation”). Increases in birth weight with the latter approach in pregnant mothers support recommendations from a 2013 narrative review (87), although the effects of the increased calories compared with animal protein per se cannot be distinguished in these interventions. The increased height in term children given balanced energy–animal protein supplementation was based on very few trials with particular characteristics: all were conducted in the 1970s, including in rural Guatemala, urban slums in Columbia, and small industrial towns in South Wales (39, 37, 38). Although our meta-analysis supports increased birth weight with balanced energy–animal protein supplementation from foods to mothers, our findings also highlight the relatively few studies testing this approach in children after birth, indicating a need for additional trials in this area.

During exclusive breastfeeding, protein accounts for ∼5% of energy intake, which generally increases to ∼15% energy when complementary foods are introduced (88). In general, protein requirements for infants and young children have been determined by the Adequate Intake method, where recommended intakes are set at the mean protein intake of healthy breastfeeding children, or ∼1.5 g/kg/d for infants 0–6 mo of age (89). These levels of intake are several-fold higher than physiologic requirements to prevent clinical amino acid deficiencies.

Yet, it remains unclear whether the amino acid requirements for body maintenance are the same as those for new tissue deposition (recovery from undernutrition) (90), and the appropriate dietary protein intake for optimal growth in children has remained uncertain. WHO has argued for inclusion of animal protein in supplementary foods in the management of moderate acute malnutrition (wasting), suggesting that “animal-source foods are more likely to meet the amino acid and other nutrient needs of recovering children” (91). However, few prior studies have systematically reviewed whether animal protein promotes optimal growth. In a recent meta-analysis, balanced energy and protein supplementation during pregnancy reduced stillbirth by 40% (95% CI: 6, 61%) and SGA by 21% (95% CI: 10, 31%), and increased birth weight by 0.04 kg (95% CI: 0.005, 0.08 kg) (92). Conversely, based on 2 trials of isocaloric-protein supplementation during pregnancy, no benefits on birth outcomes were identified; effects of either of these approaches on linear growth after birth were not evaluated (92). Another meta-analysis focused on balanced protein-energy supplementation and birth outcomes, but without differentiating plant compared with animal sources (93). Supplementation significantly increased birth weight, but not birth length or birth head circumference; again, effects on linear growth after birth were not evaluated. Consistent with our quantitative results, a recent narrative review of lipid compared with grain-based supplemental foods in the management of moderate wasting concluded that “benefits of dairy in Ready-to-Use-Food require further investigation” (94). Our finding significantly extends and expands these results by investigating animal protein supplementation during pregnancy, in preterm infants, and in term infants/children; evaluating linear growth after birth; and formally considering heterogeneity by a range of underlying characteristics.

Our systematic review also highlights the variation in the populations studied, and the nature and doses of supplementation strategies. Continuing knowledge gaps are identified because trials have used varying kinds of foods, types and levels of protein, and forms of intervention, all of which continue to make a clear articulation of the role of animal-source proteins on nutrition outcomes challenging. The differences between food-based and formula-based interventions that we identified could indicate an effect of the overall food, although chance or other design and population difference could also explain these findings. Our results highlight the need for further studies of this question, including trials concurrently testing isocaloric animal protein, animal protein including its calories, and animal foods. The heterogeneity identified in our meta-analysis as well as prior reviews indicates a need for more standardized approaches to evaluate specific forms and doses of animal protein, repeated episodes of growth failure (wasting as well as linear growth retardation), and, of course, the role of aggravating factors beyond diet. Several recently completed or ongoing trials are testing various forms and levels of animal-source foods in products specifically designed to treat acute or severe wasting in children in low-income countries. As demonstrated by our current investigation, there can be wide variability in prevalence of wasting or stunting among such populations at baseline. Among term children, we found that formula-based supplementation had larger effects on weight and height in populations with lower baseline nutritional status, the at-risk group that would drive such interventions. These same groups consistently experience greater benefits on growth in studies of pregnant women or food-based supplementation in children. These interactions by baseline nutritional status are biologically plausible and policy-relevant. Of note, however, is that few studies document the background quantity or quality (completeness) of usual total protein intake at baseline. Relatively modest differences in baseline characteristics of undernutrition, background diets, and corresponding doses and durations of supplementation could have meaningful impacts on effectiveness.

Our investigation has several strengths. Extensive searches of multiple databases, hand searching of citations, and searches of electronically linked studies reduce the likelihood that major studies corresponding to our inclusion criteria were missed. Strict inclusion criteria and duplicate data extraction reduced the possibilities of error and bias. Plausible sources of heterogeneity and the potential for small-study effects were quantitatively evaluated. Studies were identified across a range of countries, increasing generalizability. We evaluated supplementation in mothers, preterm infants, and term infants/young children, providing a more complete picture of effects across the early life course. We focused on randomized controlled trials, increasing inference for a cause-and-effect relation. Strengths of our investigation include the evaluation of different related outcomes (e.g., weight, WAZ, height, HAZ), allowing assessment for concordance of findings and elucidating robustness of the results, as well as evaluation of prespecified sources of heterogeneity to identify potential reasons for differences across studies. The observed unexplained discrepancies (e.g., for weight compared with WAZ for formula supplementation) suggest that further research is needed to confirm the effects of animal protein and animal-source foods on these outcomes.

Potential limitations should be considered. As with any meta-analysis, results are limited by the availability of studies focusing on specific outcomes of interest; for instance, relatively few studies evaluated age-specific growth outcomes, wasting, or stunting assessed using different metrics. Although we evaluated several potential sources of heterogeneity, other unknown sources, including the possibility of chance, remained. In terms of quality, many published papers evaluated using Cochrane criteria for risks of bias demonstrated elements that were either “unclear” or of relatively low rigor. This argues for much more attention to study quality going forward. For some outcomes, the small numbers and sizes of identified trials limited statistical power. On the other hand, this is the largest meta-analysis on this topic to date, serving to highlight the specific gaps in information for certain outcomes of concern.

In summary, this meta-analysis summarizes available evidence for the role of animal protein supplementation on birth, weight, and linear growth outcomes early in life. Supplementation during pregnancy and infancy/childhood increased child weight and food-based supplementation in childhood increased WAZ and reduced the risk of stunting. Only supplementation of animal-source foods (but not formula) during infancy/childhood increased height for age. Benefits on growth outcomes in preterm infants were not identified. Overall, too few high-quality studies were identified to allow for definitive conclusions on linear growth. This does not negate the benefits of balanced (protein plus energy) supplementation for recovery from wasting in children of undernourished mothers or the consideration of other potential benefits, such as on cognitive outcomes, which require separate assessment. WHO continues to support its 2012 call for use of animal-source protein in food supplements intended to manage existing malnutrition. As such, many argue that protein quality (including defined as deriving from animal-source foods) “is important to child health and not a fallacy” (95). We find that convincing evidence from multiple sources pointing to significant benefits beyond weight remains elusive and is therefore a priority for future policy-relevant research.

## Supplementary Material

nqy348_Supplemental_File

## Notes

This work was supported by a grant from the Bill & Melinda Gates Foundation (PI-Mozaffarian). CD was supported in part by the NIH K24 DK104676; and PW, in part by USAID award AID-OAA-L-10-00006 through the Feed the Future Innovation Lab for Nutrition. The funders had no role in the design and conduct of this study; collection, management, analysis, and interpretation of the data; or preparation, review, or approval of this manuscript.

Supplemental Table 1, Supplemental Figures 1–13 and Supplemental Information 1 are available from the “Supplementary data” link in the online posting of the article and from the same link in the online table of contents at https://academic.oup.com/ajcn/.

Abbreviations used: HAZ, height-for-age *z*-score; LBW, low birth weight; SGA, small for gestational age; WAZ, weight-for-age *z*-score; WFL, weight-for-length *z*-score; WMD, weighted mean difference.
