# Supplementary material for: Effects of animal protein supplementation of mothers, preterm infants, and term infants on growth outcomes in childhood: a systematic review and meta-analysis of randomized trials
Source: Am J Clin Nutr. 2019 Jun 8;110(2):410–29. doi: 10.1093/ajcn/nqy348 (PMC6669064; doi:10.1093/ajcn/nqy348)
Supplement: nqy348_Supplemental_File [file nqy348_supplemental_file.docx]

**Effects of animal protein supplementation of mothers, pre-term infants, and term infants on growth outcomes in childhood: a systematic review and meta-analysis of randomized trials**

Laura Pimpin, Sarah Kranz, Enju Liu, Masha Shulkin, Dimitra Karageorgou, Victoria Miller, Wafaie Fawzi, Christopher Duggan, Dariush Mozaffarian

**Supplemental Material 1:**

A total of 62 randomized controlled trials with 86 separate interventions arms met eligibility criteria, totaling 30,349 unique participants. (1-62)

**Supplemental Material 2:**

Electronic searches

### Cochrane library

1. child or children or infant or newborn or pregnancy or pregnant or maternal or prenatal or baby or babies or boy or girl or toddler or preschool or pre-school:ti,ab,kw (Word variations have been searched)
2. dietary protein or protein intake or animal protein or meat or egg or dairy or milk or fish or seafood or "protein deficiency" or "protein-energy malnutrition" or malnutrition:ti,ab,kw (Word variations have been searched)
3. height or length or growth or stunting or birthweight or "birth weight" or "small for gestational age" or body weight or body size or "pregnancy outcome" or anthropometry or anthropometrics or weight gain or body composition or premature or "intrauterine growth" or "fetal growth":ti,ab,kw (Word variations have been searched)
4. 1 AND 2 AND 3

**n= 125 cochrane reviews + 70 in 2018 update = 195**

### Pubmed

((((dietary protein[MeSH Terms] OR meat[ MeSH Terms] OR dairy products[MeSH Terms] OR eggs[MeSH Terms] OR dietary supplements[MeSH Terms] OR food, fortified[MeSH Terms] OR energy intake[MeSH Terms] OR “dietary protein”[Title/Abstract] OR “protein supplement*”[Title/Abstract]) AND (body height[MeSH Terms] OR growth[MeSH Terms] OR growth disorders[MeSH Terms] OR Child Nutrition Disorders[MeSH Terms] OR Child Development[MeSH Terms] OR Pregnancy Outcome[MeSH Terms] OR Birth Weight[MeSH Terms] OR Premature Birth[MeSH Terms] OR Infant, Small for Gestational Age[MeSH Terms] OR Small for Gestational Age[Title/Abstract] OR birth weight[Title/Abstract] OR stunting[Title/Abstract] OR height[Title/Abstract]) AND (child[MeSH Terms] OR infant[MeSH Terms] OR pregnancy[MeSH Terms] OR pregnant women[MeSH Terms] OR children[Title/Abstract] OR child[Title/Abstract] OR infants[Title/Abstract] OR infant[Title/Abstract] OR pregnant[Title/Abstract] OR pregnancy[Title/Abstract] OR maternal[Title/Abstract] OR prenatal[Title/Abstract]) AND (clinical trial[MeSH Terms] OR randomized controlled trial[MeSH Terms] OR meta analysis[MeSH Terms] OR cohort studies[MeSH Terms] OR longitudinal studies[MeSH Terms] OR random allocation[MeSH Terms] OR clinical trial[Publication Type] OR controlled clinical trial[Publication Type] OR randomized controlled trial[Publication Type] OR meta analysis[Publication Type]))

**N=3565 + 883 in 2018 update = 4448**

Web of Science

(TS=("dietary protein" OR "protein intake" OR "animal protein" OR meat OR egg OR dairy OR milk OR fish OR seafood OR "protein deficiency" OR "protein-energy malnutrition" OR malnutrition)) AND (TS=( height OR length OR growth OR stunting OR birthweight OR "birth weight" OR "small for gestational age" OR "body weight" OR "body size" OR "pregnancy outcome*" OR anthropometry OR anthropometrics OR weight gain OR "body composition" OR premature ))AND (TI=("trial*" OR "randomization" OR "randomized" OR "intervention*" OR "prospective study" OR "longitudinal study" OR "cohort" OR " follow-up" OR "follow up" ))AND (TS=( child OR children OR infant OR newborn OR pregnancy OR pregnant OR maternal OR prenatal OR baby OR babies OR boy OR girl OR toddler OR preschool OR pre-school ))

### Indexes=SCI-EXPANDED, CPCI-S, CPCI-SSH, BKCI-S, BKCI-SSH Timespan=All years

**N=995 + 574 in 2018 update = 1569**

### Ovid EMBASE

1 exp *protein intake/ (13231)

2 height/ or body height/ (70383)

3 exp birth weight/ (81622)

4 exp child growth/ or exp postnatal growth/ (12304)

5 child/ (1321806)

6 infant/ (522739)

7 child/ (1321806)

8 (child or infant).mp. [mp=title, abstract, subject headings, heading word, drug trade name, original title, device manufacturer, drug manufacturer, device trade name, keyword] (2064959)

9 2 or 3 or 4 (154698)

10 1 and 8 and 9 (191)

11 exp diet supplementation/ (65880)

12 named groups by pregnancy/ or exp "named groups of persons"/ (3082555)

13 from 10 keep 1-191 (191)

14 exp pregnant woman/ (36111)

15 1 or 11 (78256)

16 8 or 14 (2092812)

17 9 and 15 and 16 (1570)

18 controlled clinical trial/ or clinical trial/ or exp randomized controlled trial/ (942618)

19 exp cohort analysis/ (186481)

20 exp longitudinal study/ (71923)

21 18 or 19 or 20 (1174562)

22 17 and 21

**N= 530 +148 in 2018 update = 678**

### CINAHL

( (MH "Dietary Proteins+") OR (MH Meat+) OR (MH“Dairy Products+”) OR (MH Eggs+) OR (MH "Dietary Supplements+") OR (MH "Food, fortified+") OR TI "Dietary Protein" OR AB "Dietary Protein" OR TI "Protein supplement*" OR AB "Protein supplement*" OR (MH “Energy Intake+”) ) AND ( TI stunting OR AB stunting OR (MH “Body Height+”) OR TI height OR AB height OR (MH “Growth+”) OR (MH “Growth Disorders+”) OR (MH “Child Nutrition Disorders+”) OR (MH “Child Development+”) OR (MH “Pregnancy Outcome+”) OR (MH “Birth weight+”) OR (MH “Premature Birth+”) OR (MH “Small for gestational age+”) OR TI “Birth weight” OR AB “Birth weight” OR TI “small for gestational age” OR AB “small for gestational age” ) AND ( TI child OR AB child OR TI children OR AB children OR TI infant OR AB infant OR TI pregnant OR AB pregnant OR TI pregnancy OR AB pregnancy OR TI maternal OR AB maternal OR TI prenatal OR AB prenatal OR (MH “Child+”) OR (MH “Infant+”) OR (MH “Pregnancy+”) OR (MH “Pregnant Women+”) ) AND ( (MH “Clinical Trials+”) OR (MH “Meta Analysis+”) OR (MH “Prospective Studies+”) )

**N=569 + 150 in 2018 update = 719**

### LILACS

((mh:(dietary proteins)) OR (tw:(dietary protein)) OR (tw:(proteina)) OR (tw:(protein))) AND

((tw:(growth)) OR (tw:(body height)) OR (tw:(crecimiento)) OR (tw:(crescimiento)) OR (tw:(estatura)) OR (tw:(birthweight)) OR (tw:(birth weight)) OR (tw:(peso al nacer)) OR (tw:(peso ao nacer)) OR (tw:(premature)) OR (tw:(prematuro)) OR (mh:(growth)) OR (mh:(body height)) OR (mh:(birth weight)) OR (mh:(infant, low birthweight))) AND ((mh:(child)) OR (mh:(infant)) OR (mh:(Pregnant women)) OR (tw:(child)) OR (tw:(nino)) OR (tw:(criança)) OR (tw:(infant)) OR (tw:(lacatante)) OR (tw:(lactente)) OR (tw:(pregnant women)) OR (tw:(mujeres embarazadas)) OR (tw:(gestantes)))

**N=428 + 46 in 2018 update =474**

**References:**

1. Ackatia-Armah RS, McDonald CM, Doumbia S, Erhardt JG, Hamer DH, Brown KH. Malian children with moderate acute malnutrition who are treated with lipid-based dietary supplements have greater weight gains and recovery rates than those treated with locally produced cereal-legume products: a community-based, cluster-randomized trial. Am J Clin Nutr. 2015;101(3):632-45.

2. Adams SO, Barr GD, Huenemann RL. Effect of nutritional supplementation in pregnancy. I. Outcome of pregnancy. Journal of the American Dietetic Association. 1978;72(2):144-7.

3. Adu-Afarwuah S, Lartey A, Okronipa H, Ashorn P, Zeilani M, Peerson JM, et al. Lipid-based nutrient supplement increases the birth size of infants of primiparous women in Ghana. Am J Clin Nutr. 2015;101(4):835-46.

4. Aimone A, Rovet J, Ward W, Jefferies A, Campbell DM, Asztalos E, et al. Growth and body composition of human milk-fed premature infants provided with extra energy and nutrients early after hospital discharge: 1-year follow-up. Journal of pediatric gastroenterology and nutrition. 2009;49(4):456-66.

5. Alarcon PA, Lin LH, Noche M, Jr., Hernandez VC, Cimafranca L, Lam W, et al. Effect of oral supplementation on catch-up growth in picky eaters. Clinical pediatrics. 2003;42(3):209-17.

6. Amesz EM, Schaafsma A, Cranendonk A, Lafeber HN. Optimal growth and lower fat mass in preterm infants fed a protein-enriched postdischarge formula. Journal of pediatric gastroenterology and nutrition. 2010;50(2):200-7.

7. Ashorn P, Alho L, Ashorn U. Supplementation of Maternal Diets during Pregnancy and for 6 Months Postpartum and Infant Diets Thereafter with Small-Quantity Lipid-Based Nutrient Supplements Does Not Promote Child Growth by 18 Months of Age in Rural Malawi: A Randomized Controlled Trial. 2015;145(6):1345-53.

8. Bauserman M, Lokangaka A, Gado J, Close K, Wallace D, Kodondi K-K, et al. A cluster-randomized trial determining the efficacy of caterpillar cereal as a locally available and sustainable complementary food to prevent stunting and anaemia. Public health nutrition. 2015;18(10):1785-92.

9. Borschel MW, Ziegler EE, Wedig RT, Oliver JS. Growth of healthy term infants fed an extensively hydrolyzed casein-based or free amino acid-based infant formula: a randomized, double-blind, controlled trial. Clinical pediatrics. 2013;52(10):910-7.

10. Carver JD, Wu PY, Hall RT, Ziegler EE, Sosa R, Jacobs J, et al. Growth of preterm infants fed nutrient-enriched or term formula after hospital discharge. Pediatrics. 2001;107(4):683-9.

11. Chan GM, McElligott K, McNaught T, Gill G. Effects of dietary calcium intervention on adolescent mothers and newborns: A randomized controlled trial. Obstetrics and gynecology. 2006;108(3 Pt 1):565-71.

12. Christian P, Shaikh S, Shamim AA, Mehra S, Wu L, Mitra M, et al. Effect of fortified complementary food supplementation on child growth in rural Bangladesh: a cluster-randomized trial. International journal of epidemiology. 2015;44(6):1862-76.

13. Cooke RJ, Griffin IJ, McCormick K. Adiposity is not altered in preterm infants fed with a nutrient-enriched formula after hospital discharge. Pediatric research. 2010;67(6):660-4.

14. Elwood PC, Haley TJ, Hughes SJ, Sweetnam PM, Gray OP, Davies DP. Child growth (0-5 years), and the effect of entitlement to a milk supplement. Archives of disease in childhood. 1981;56(11):831-5.

15. Embleton ND, Cooke RJ. Protein requirements in preterm infants: effect of different levels of protein intake on growth and body composition. Pediatric research. 2005;58(5):855-60.

16. Fabiansen C, Yaméogo CW, Iuel-Brockdorf A-S, Cichon B, Rytter MJ, Kurpad A, et al. Effectiveness of food supplements in increasing fat-free tissue accretion in children with moderate acute malnutrition: A randomised 2× 2× 3 factorial trial in Burkina Faso. PLoS medicine. 2017;14(9):e1002387.

17. Fazzolari-Nesci A, Domianello D, Sotera V, Raiha NC. Tryptophan fortification of adapted formula increases plasma tryptophan concentrations to levels not different from those found in breast-fed infants. Journal of pediatric gastroenterology and nutrition. 1992;14(4):456-9.

18. Fleddermann M, Demmelmair H, Grote V, Nikolic T, Trisic B, Koletzko B. Infant formula composition affects energetic efficiency for growth: the BeMIM study, a randomized controlled trial. Clinical nutrition (Edinburgh, Scotland). 2014;33(4):588-95.

19. Fomon SJ, Ziegler EE, Nelson SE, Frantz JA. What is the safe protein-energy ratio for infant formulas? Am J Clin Nutr. 1995;62(2):358-63.

20. Graham GG, MacLean WC, Jr., Brown KH, Morales E, Lembcke J, Gastanaduy A. Protein requirements of infants and children: growth during recovery from malnutrition. Pediatrics. 1996;97(4):499-505.

21. Hanning RM, Paes B, Atkinson SA. Protein metabolism and growth of term infants in response to a reduced-protein, 40:60 whey: casein formula with added tryptophan. Am J Clin Nutr. 1992;56(6):1004-11.

22. He M, Yang YX, Han H, Men JH, Bian LH, Wang GD. Effects of yogurt supplementation on the growth of preschool children in Beijing suburbs. Biomedical and environmental sciences : BES. 2005;18(3):192-7.

23. Heikens GT, Schofield WN, Dawson S. The Kingston Project. II. The effects of high energy supplement and metronidazole on malnourished children rehabilitated in the community: anthropometry. European journal of clinical nutrition. 1993;47(3):160-73.

24. Heikens GT, Schofield WN, Dawson S, Grantham-McGregor S. The Kingston project. I. Growth of malnourished children during rehabilitation in the community, given a high energy supplement. European journal of clinical nutrition. 1989;43(3):145-60.

25. Iannotti LL, Lutter CK, Stewart CP, Riofrío CAG, Malo C, Reinhart G, et al. Eggs in early complementary feeding and child growth: a randomized controlled trial. Pediatrics. 2017:e20163459.

26. Kardjati S, Kusin JA, De With C. Energy supplementation in the last trimester of pregnancy in East Java: I. Effect on birthweight. British journal of obstetrics and gynaecology. 1988;95(8):783-94.

27. Koletzko B, von Kries R, Closa R, Escribano J, Scaglioni S, Giovannini M, et al. Lower protein in infant formula is associated with lower weight up to age 2 y: a randomized clinical trial. Am J Clin Nutr. 2009;89(6):1836-45.

28. Koo WW, Hockman EM. Posthospital discharge feeding for preterm infants: effects of standard compared with enriched milk formula on growth, bone mass, and body composition. Am J Clin Nutr. 2006;84(6):1357-64.

29. Krebs NF, Mazariegos M, Chomba E, Sami N, Pasha O, Tshefu A, et al. Randomized controlled trial of meat compared with multimicronutrient-fortified cereal in infants and toddlers with high stunting rates in diverse settings. Am J Clin Nutr. 2012;96(4):840-7.

30. Larnkjaer A, Hoppe C, Molgaard C, Michaelsen KF. The effects of whole milk and infant formula on growth and IGF-I in late infancy. European journal of clinical nutrition. 2009;63(8):956-63.

31. Lien EL, Davis AM, Euler AR. Growth and safety in term infants fed reduced-protein formula with added bovine alpha-lactalbumin. Journal of pediatric gastroenterology and nutrition. 2004;38(2):170-6.

32. Lin CA, Manary MJ, Maleta K, Briend A, Ashorn P. An energy-dense complementary food is associated with a modest increase in weight gain when compared with a fortified porridge in Malawian children aged 6-18 months. The Journal of nutrition. 2008;138(3):593-8.

33. Long JK, Murphy SP, Weiss RE, Nyerere S, Bwibo NO, Neumann CG. Meat and milk intakes and toddler growth: a comparison feeding intervention of animal-source foods in rural Kenya. Public health nutrition. 2012;15(6):1100-7.

34. Lonnerdal B, Chen CL. Effects of formula protein level and ratio on infant growth, plasma amino acids and serum trace elements. I. Cow's milk formula. Acta paediatrica Scandinavica. 1990;79(3):257-65.

35. Lonnerdal B, Hernell O. Effects of feeding ultrahigh-temperature (UHT)-treated infant formula with different protein concentrations or powdered formula, as compared with breast-feeding, on plasma amino acids, hematology, and trace element status. Am J Clin Nutr. 1998;68(2):350-6.

36. Maleta KM, Phuka J, Alho L, Cheung YB, Dewey KG, Ashorn U, et al. Provision of 10–40 g/d Lipid-Based Nutrient Supplements from 6 to 18 Months of Age Does Not Prevent Linear Growth Faltering in Malawi–3. The Journal of nutrition. 2015;145(8):1909-15.

37. Mangani C, Maleta K, Phuka J, Cheung YB, Thakwalakwa C, Dewey K, et al. Effect of complementary feeding with lipid-based nutrient supplements and corn-soy blend on the incidence of stunting and linear growth among 6- to 18-month-old infants and children in rural Malawi. Matern Child Nutr. 2015;11 Suppl 4:132-43.

38. Mardones-Santander F, Rosso P, Stekel A, Ahumada E, Llaguno S, Pizarro F, et al. Effect of a milk-based food supplement on maternal nutritional status and fetal growth in underweight Chilean women. Am J Clin Nutr. 1988;47(3):413-9.

39. Mora JO, Herrera MG, Suescun J, de Navarro L, Wagner M. The effects of nutritional supplementation on physical growth of children at risk of malnutrition. Am J Clin Nutr. 1981;34(9):1885-92.

40. Mridha MK, Matias SL, Chaparro CM, Paul RR, Hussain S, Vosti SA, et al. Lipid-based nutrient supplements for pregnant women reduce newborn stunting in a cluster-randomized controlled effectiveness trial in Bangladesh. Am J Clin Nutr. 2016;103(1):236-49.

41. Nikiema L, Huybregts L, Kolsteren P, Lanou H, Tiendrebeogo S, Bouckaert K, et al. Treating moderate acute malnutrition in first-line health services: an effectiveness cluster-randomized trial in Burkina Faso. Am J Clin Nutr. 2014;100(1):241-9.

42. Oropeza-Ceja L, Rosado J, Ronquillo D, García O, Caamaño M, García-Ugalde C, et al. Lower Protein Intake Supports Normal Growth of Full-Term Infants Fed Formula: A Randomized Controlled Trial. Nutrients. 2018;10(7):886.

43. Raiha NC, Fazzolari-Nesci A, Cajozzo C, Puccio G, Monestier A, Moro G, et al. Whey predominant, whey modified infant formula with protein/energy ratio of 1.8 g/100 kcal: adequate and safe for term infants from birth to four months. Journal of pediatric gastroenterology and nutrition. 2002;35(3):275-81.

44. Rush D, Stein Z, Susser M. A randomized controlled trial of prenatal nutritional supplementation in New York City. Pediatrics. 1980;65(4):683-97.

45. Rzehak P, Sausenthaler S, Koletzko S, Reinhardt D, von Berg A, Kramer U, et al. Short- and long-term effects of feeding hydrolyzed protein infant formulas on growth at < or = 6 y of age: results from the German Infant Nutritional Intervention Study. Am J Clin Nutr. 2009;89(6):1846-56.

46. Schlossman N, Brown C, Batra P, de Sa AB, Balan I, Balan A, et al. A randomized controlled trial of two ready-to-use supplementary foods demonstrates benefit of the higher dairy supplement for reduced wasting in mothers, and differential impact in infants and children associated with maternal supplement response. Food Nutr Bull. 2017;38(3):275-90.

47. Schmelzle H, Wirth S, Skopnik H, Radke M, Knol J, Bockler HM, et al. Randomized double-blind study of the nutritional efficacy and bifidogenicity of a new infant formula containing partially hydrolyzed protein, a high beta-palmitic acid level, and nondigestible oligosaccharides. Journal of pediatric gastroenterology and nutrition. 2003;36(3):343-51.

48. Schroeder DG, Martorell R, Rivera JA, Ruel MT, Habicht JP. Age differences in the impact of nutritional supplementation on growth. The Journal of nutrition. 1995;125(4 Suppl):1051s-9s.

49. Simondon KB, Gartner A, Berger J, Cornu A, Massamba JP, San Miguel JL, et al. Effect of early, short-term supplementation on weight and linear growth of 4-7-mo-old infants in developing countries: a four-country randomized trial. Am J Clin Nutr. 1996;64(4):537-45.

50. Skau JK, Touch B, Chhoun C, Chea M, Unni US, Makurat J, et al. Effects of animal source food and micronutrient fortification in complementary food products on body composition, iron status, and linear growth: a randomized trial in Cambodia. Am J Clin Nutr. 2015;101(4):742-51.

51. Stobaugh HC, Ryan KN, Kennedy JA, Grise JB, Crocker AH, Thakwalakwa C, et al. Including whey protein and whey permeate in ready-to-use supplementary food improves recovery rates in children with moderate acute malnutrition: a randomized, double-blind clinical trial. Am J Clin Nutr. 2016;103(3):926-33.

52. Tang M, Sheng XY, Krebs NF, Hambidge KM. Meat as complementary food for older breastfed infants and toddlers: a randomized, controlled trial in rural China. Food Nutr Bull. 2014;35(4 Suppl):S188-92.

53. Tang MH, Krebs NF. High protein intake from meat as complementary food increases growth but not adiposity in breastfed infants: a randomized trial. American Journal of Clinical Nutrition. 2014;100(5):1322-8.

54. Tavill F, Gonik A. Use of fish protein concentrate in the diets of weanling infants. A study of 88 infants from a low socioeconomic population, Casablanca, Morocco. Am J Clin Nutr. 1969;22(12):1571-6.

55. Timby N, Domellof E, Hernell O, Lonnerdal B, Domellof M. Neurodevelopment, nutrition, and growth until 12 mo of age in infants fed a low-energy, low-protein formula supplemented with bovine milk fat globule membranes: a randomized controlled trial. Am J Clin Nutr. 2014;99(4):860-8.

56. Turck D, Grillon C, Lachambre E, Robiliard P, Beck L, Maurin JL, et al. Adequacy and safety of an infant formula with a protein/energy ratio of 1.8 g/100 kcal and enhanced protein efficiency for term infants during the first 4 months of life. Journal of pediatric gastroenterology and nutrition. 2006;43(3):364-71.

57. Viegas OA, Scott PH, Cole TJ, Eaton P, Needham PG, Wharton BA. Dietary protein energy supplementation of pregnant Asian mothers at Sorrento, Birmingham. II: Selective during third trimester only. British medical journal (Clinical research ed). 1982;285(6342):592-5.

58. Walker SP, Grantham-McGregor SM, Himes JH, Powell CA, Chang SM. Early childhood supplementation does not benefit the long-term growth of stunted children in Jamaica. The Journal of nutrition. 1996;126(12):3017-24.

59. Weber M, Grote V, Closa-Manesterolo R, Escribano J, Langhendries JP, Dain E, et al. Lower protein content in infant formula reduces BMI and obesity risk at school age: follow-up of a randomized trial. American Journal of Clinical Nutrition. 2014;99(5):1041-51.

60. Wohlleb JC, Pollitt E, Mueller WH, Bigelow R. The Bacon Chow study: maternal supplementation and infant growth. Early human development. 1983;9(1):79-91.

61. Ziegler EE, Fields DA, Chernausek SD, Steenhout P, Grathwohl D, Jeter JM, et al. Adequacy of infant formula with protein content of 1.6 g/100 kcal for infants between 3 and 12 months. Journal of pediatric gastroenterology and nutrition. 2015;61(5):596-603.

62. Ziegler EE, Jeter JM, Drulis JM, Nelson SE, Haschke F, Steenhout P, et al. Formula with reduced content of improved, partially hydrolyzed protein and probiotics: Infant growth and health. Monatsschrift fur Kinderheilkunde. 2003;151(SUPPL. 1):S65-S71.

**Supplemental Figure 1 – PRISMA Flowchart of study selection and inclusion**


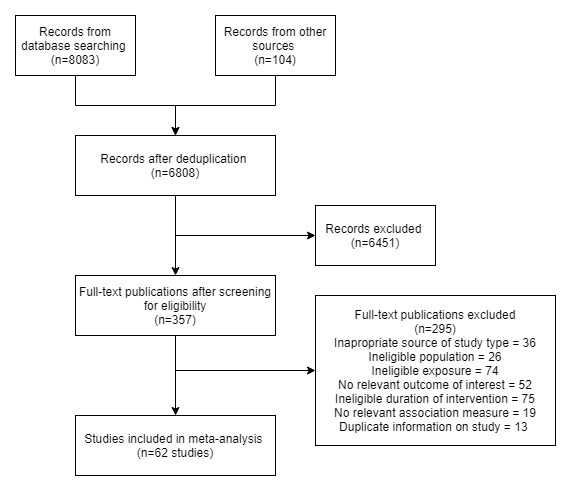


**Supplemental Table 1. Study Quality Score According to Cochrane Risk of Bias Criteria**

| **Study Author/Date** | **Risk of inadequate generation of a randomized sequence** | **Risk of inadequate concealment of allocation prior to assignment** | **Knowledge of allocated intervention by participants and personnel during the study** | **Knowledge of allocated intervention by outcome assessors** | **Inadequate assessment of incomplete outcome data** | **Suggestion of selective outcome reporting** |
| --- | --- | --- | --- | --- | --- | --- |
| **Pregnant Mothers** | | | | | | |
| Adams 1978 | Unclear | Unclear | Unclear | Unclear | Unclear | Low |
| Adu-Afarwuah 2015 | Low | Low | Low | Low | Low | Low |
| Chan 2006 | Unclear | High | High | High | Unclear | Low |
| Kardjati 1989 | High | High | High | Low | Unclear | Low |
| Mardones-Santander 1991 | Low | Low | Low | Low | Low | Low |
| Mridha 2016 | Low | Low | Low | Low | Low | Low |
| Rush 1980 | Unclear | High | High | Unclear | Unclear | Low |
| Viegas 1982 | High | Unclear | Unclear | Unclear | High | Low |
| Wohlleb 1983 | Low | Unclear | Unclear | Unclear | Unclear | Low |
| **Pregnant Mother and Offspring** | | | | | | |
| Ashorn 2015 | Low | Low | Low | Low | Low | Low |
| Elwood 1981 | Unclear | High | High | High | Unclear | Low |
| Mora 1981 | Unclear | Unclear | Unclear | Unclear | Unclear | Low |
| Schroeder 1995 | High | Unclear | Unclear | Unclear | High | Low |
| **Preterm Children** | | | | | | |
| Aimone 2009 | Low | High | High | Low | Low | Low |
| Amesz 2010 | Unclear | High | High | High | Unclear | Low |
| Carver 2001 | Low | Unclear | Low | Low | Low | Low |
| Cooke 2010 | Unclear | High | High | High | Unclear | Low |
| Embleton 2005 | Unclear | High | High | High | Unclear | Low |
| Koo 2006 | Low | Low | Low | Low | Low | Low |
| **Term Children (Formula Interventions)** | | | | | | |
| Borschel 2013 | Unclear | High | High | High | Unclear | Low |
| Fazzolari-Nesci 1992 | Unclear | Unclear | Low | Unclear | Unclear | Low |
| Fledderman 2014 | Low | Low | Low | Low | Low | Low |
| Fomon 1995 | Unclear | High | High | Unclear | Unclear | Low |
| Graham 1996 | Low | Low | Low | Low | Low | Unclear |
| Hanning 1992 | Low | Low | High | Unclear | Low | Low |
| Koletzko 2009 | Low | Low | Low | Low | Low | Low |
| Larnkjaer 2009 | Low | Low | Low | Low | Low | Low |
| Lien 2004 | Low | Low | Low | Unclear | Low | Low |
| Lonnerdal 1990 | Low | Low | Low | Low | Low | Low |
| Lonnerdal 1998 | Unclear | Unclear | Low | Unclear | Low | Low |
| Oropeza-Ceja 2018 | Low | Low | Low | Low | Low | Low |
| Raiha 2002 | Unclear | Unclear | Unclear | Unclear | Unclear | Low |
| Rzehak 2009 | High | Unclear | Unclear | Unclear | High | Low |
| Schmelzle 2003 | Low | Unclear | Low | Unclear | Low | Low |
| Timby 2014 | High | Unclear | Unclear | Unclear | High | Low |
| Turck 2006 | High | Unclear | Unclear | Unclear | High | Low |
| Weber 2014 | High | Unclear | Unclear | Unclear | High | Low |
| Ziegler 2003 | Unclear | Unclear | Unclear | Unclear | Unclear | Low |
| Ziegler 2015 | Low | Unclear | Low | Low | Low | Low |
| **Term Children (Whole Food Intervention)** | | | | | | |
| Ackatia-Armah 2015 | Low | Unclear | Unclear | Unclear | Low | Low |
| Alarcon 2003 | Unclear | High | High | High | Unclear | Low |
| Bauserman 2015 | Low | Low | High | Low | Low | Low |
| Christian 2015 | Low | High | High | High | Low | Low |
| Fabiansen 2017 | Low | Low | Low | Low | Low | Unclear |
| He 2005 | Low | Low | Unclear | Unclear | Low | Low |
| Heikens 1989 | Unclear | High | High | Unclear | Low | Low |
| Heikens 1993 | High | High | High | Low | Unclear | Low |
| Iannotti 2017 | Low | Low | High | Low | Low | Low |
| Krebs 2012 | Low | Low | Low | Low | Low | Low |
| Lin 2008 | Low | High | High | Unclear | Low | Low |
| Long 2012 | Low | Low | Low | Low | Low | Low |
| Maleta 2015 | Low | Low | Low | Low | High | Low |
| Mangani 2015 | Low | Low | Low | Low | Low | Low |
| Nikiema 2014 | Low | High | High | Unclear | Low | Low |
| Schlossman 2017 | Low | Low | Low | Unclear | High | Low |
| Simondon 1996 | Low | Unclear | Unclear | Unclear | Low | Low |
| Skau 2015 | Low | Low | Low | High | Low | Low |
| Stobaugh 2016 | Low | Low | Low | Low | Low | Low |
| Tang 2014 | High | Unclear | Unclear | Unclear | High | Low |
| Tang 2014 | High | Unclear | Unclear | Unclear | High | Low |
| Tavill 1969 | High | Unclear | Unclear | Unclear | High | Low |
| Walker 1996 | High | Unclear | Unclear | Unclear | High | Low |

**Supplemental Figure 2. Effects of protein supplementation on odds of (A) low birthweight from 5 estimates from 5 trials including 6,121 subjects and (B) small for gestational age from 4 estimates from 4 trials including 5,674 subjects.**

| A   | B   |
| --- | --- |

**Supplemental Figure 3. Effects of maternal protein supplementation on (A) height in cm from 3 estimates from 3 trials including 1,490 subjects (A) and (B) weight in kg from 2 estimates from 2 trials including 636 subjects. SMD=standardized (weighted) mean difference.**

| A   | B   |
| --- | --- |

**Supplemental Figure 4. Effects of maternal and child protein supplementation on (A) height in cm from 2 estimates from 2 trials including 3,276 subjects and (B) weight in kg from 3 estimates from 3 trials including 4,227 subjects. SMD=standardized (weighted) mean difference.**

| A | B   |
| --- | --- |

**Supplemental Figure 5. Effects pre-term child protein supplementation on (A) height in cm from 5 estimates from 4 trials including 262 subjects and (B) height-for-age z-score from 4 estimates from 3 trials including 269 subjects. SMD=standardized (weighted) mean difference.**

| A   | B   |
| --- | --- |

**Supplemental Figure 6. Effects pre-term child protein supplementation on weight in kg from 6 estimates from 5 trials including 373 subjects. SMD=standardized (weighted) mean difference.**

**Supplemental Figure 7. Effects of pre-term child protein supplementation on (A) weight-for-age z-score from 2 estimates from 2 trials including 169 subjects and (B) weight-for-length z-score from 2 estimates from 1 trial including 100 subjects. SMD=standardized (weighted) mean difference.**

| A   | B   |
| --- | --- |

**Supplemental Figure 8. Effects term infant formula protein supplementation on (A) height-for-age z-score from 7 estimates from 6 trials including 1,532 subjects and (B) weight-for-age z-score from 7 estimates from 6 trials including 1,532 subjects. SMD=standardized (weighted) mean difference.**

| A   | B   |
| --- | --- |

**Supplemental Figure 9. Effects term infant formula protein supplementation on weight-for-length z-score from 3 estimates from 2 trials including 711 subjects SMD=standardized (weighted) mean difference.**

**Supplemental Figure 10. Effects child infant and child food-based protein supplementation on height-for-age z-score from 19 estimates from 15 trials including 6,608 subjects. SMD=standardized (weighted) mean difference.**

**Supplemental Figure 11: Effects of term infant/child food-based protein supplementation on (A) weight-for-age z-score from 15 estimates from 12 trials including 5,611 subjects and (B) weight-for-length z-score from 19 estimates from 15 trials including 11,251 subjects. SMD=standardized (weighted) mean difference.**

| A   | B   |
| --- | --- |

**Supplemental Figure 12: Effects of term infant/child food-based protein supplementation on odds of (A) stunting from 6 estimates from 3 trials including 5,138 subjects and (B) wasting from 5 estimates from 3 trials including 5,267 subjects.**

| A   | B   |
| --- | --- |

**Supplemental Figure 13. Evaluation of small study effects using funnel plots and Egger's and Begg’ tests. SMD=standardized (weighted) mean difference.**

| **Funnel Plots** | **P value for Egger’s test** | **P value for Begg’s test** |
| --- | --- | --- |
| Effects of maternal supplementation on birthweight in kg from 14 estimates in 12 trials including 8132 subjects. |  |  |
|  | 0.615 | 0.298 |
| Effects of term infant/child protein formula supplementation on height in cm from 24 estimates in 18 trials including 2,923 subjects. |  |  |
|  | 0.064 | 0.143 |
| Effects of term child protein formula supplementation on weight in kg from 24 estimates from 18 trials including 2,923 subjects. |  |  |
|  | 0.271 | 0.102 |
| Effects of term child protein food-based supplementation on height in cm from 25 estimates from 18trials including 13,626 subjects. |  |  |
|  | 0.317 | 0.889 |
| Effects of term child protein food-based supplementation on weight in kg from 23 estimates from 16 trials including 11,195 subjects. |  |  |
|  | 0.338 | 0.444 |
| **Effects child infant and child food-based protein supplementation on height-for-age z-score from 19 estimates from 15 trials including 6,608 subjects.** |  |  |
| **** | 0.449 | 0.463 |
| **Effects of term infant/child food-based protein supplementation on weight-for-age z-score from 15 estimates from 12 trials including 5,611 subjects** |  |  |
| **** | 0.492 | 0.690 |
| Effects of **term infant/child food-based protein supplementation** on **weight-for-length z-score from 19 estimates from 15 trials including 11,251 subjects. SMD=standardized (weighted) mean difference.** |  |  |
|  | 0.851 | 0.700 |
